# Bioinformatics analysis of C3 and CXCR4 demonstrates their potential as prognostic biomarkers in clear cell renal cell carcinoma (ccRCC)

**DOI:** 10.1186/s12885-021-08525-w

**Published:** 2021-07-15

**Authors:** Jing Quan, Yuchen Bai, Yunbei Yang, Er Lei Han, Hong Bai, Qi Zhang, Dahong Zhang

**Affiliations:** 1 Department of Urology, Zhejiang Provincial People’s Hospital, People’s Hospital of Hangzhou Medical College, Hangzhou, 310014 China; 2 Department of Cardiothoracic Surgery, Zhejiang Provincial People’s Hospital, People’s Hospital of Hangzhou Medical College, Hangzhou, 310014 China

**Keywords:** Gene, Biomarker, ccRCC, Bioinformatics analysis

## Abstract

**Background:**

The molecular prognostic biomarkers of clear cell renal cell carcinoma (ccRCC) are still unknown. We aimed at researching the candidate biomarkers and potential therapeutic targets of ccRCC.

**Methods:**

Three ccRCC expression microarray datasets (include GSE14762, GSE66270 and GSE53757) were downloaded from the gene expression omnibus (GEO) database. The differentially expressed genes (DEGs) between ccRCC and normal tissues were explored. The potential functions of identified DEGs were analyzed by Gene Ontology (GO) and Kyoto Encyclopedia of Genes and Genomes (KEGG). And then the protein - protein interaction network (PPI) was established to screen the hub genes. After that, the expressions of hub genes were identified by the oncomine database. The hub genes’ prognostic values of patients with ccRCC were analyzed by GEPIA database.

**Results:**

A total of 137 DEGs were identified by utilizing the limma package and RRA method, including 63 upregulated genes and 74 downregulated genes. It is found that 137 DEGs were mainly enriched in 82 functional terms and 24 pathways in accordance with the research results. Thirteen highest-scoring genes were screened as hub genes (include 10 upregulated genes and 3 downregulated candidate genes) by utilizing the PPI network and module analysis. Through integrating the oncoming database and GEPIA database, the author found that C3 and CXCR4 are not only overexpressed in ccRCC, but also associated with the prognosis of ccRCC. Further results could reveal that patients with high C3 expression had a poor overall survival (OS), while patients with high CTSS and TLR3 expressions had a good OS; patients with high C3 and CXCR4 expressions had a poor disease-free survival (DFS), while ccRCC patients with high TLR3 expression had a good DFS.

**Conclusion:**

These findings suggested that C3 and CXCR4 were the candidate biomarkers and potential therapeutic targets of ccRCC patients.

**Supplementary Information:**

The online version contains supplementary material available at 10.1186/s12885-021-08525-w.

## Introduction

Renal cell carcinoma (RCC) is the most common kidney malignancies, which originates in the renal tubular epithelium [1]. Among of RCC, clear cell RCC (ccRCC) is the most important histological subtype, accounting for ∼80% of RCC [2]. The vast majority of RCC are discovered by accident. Less than 5% of RCC are detected by the classic triad (gross hematuria, flank pain and abdominal mass) and are often advanced. Due to resistant to radiotherapy and chemotherapy, surgical resection is still the optimal treatment for RCC [2]. Although the emergence of immunotherapy and targeted therapy has diversified the treatment of RCC, the prognosis of patients with RCC who have lost the opportunity of surgery remains dismal [3]. Therefore, it is particularly important to understand the pathogenesis of RCC and investigate biomarkers to support the treatment and prediction of prognosis.

In recent years, bioinformatics analysis of gene expression microarrays could help identify the potential target genes of diseases and provide the molecular characteristics, regulatory pathways and cellular networks of diseases [4]. The gene expression omnibus (GEO, www.ncbi.nlm.nih.gov/geo/) database is an international public functional genomics database, which stores common array and sequence data. In the past decades, more and more scholars had indicated that tumor-related genes were discovered by using GEO databases in their researches. For instance, Guo et al. found that 31 mostly changed hub genes were significant enriched in several pathways through integrated bioinformatical analysis, which mainly associated with cell cycle process, chemokines and G-protein coupled receptor signaling pathways in colorectal cancer [5]. Besides, Liang’s research results indicated that BCL2, CCND1 and COL1A1 might be the key genes in thyroid papillary carcinoma through bioinformatics analysis [6]. What’s more, bioinformatics has been widely used in the diagnosis and prognosis of renal cancer. For example, li et al. found that MMP2, DCN, COL4A1, CASR, GPR4, UTS2, and LDLR can be regarded as potential immunotherapy biomarkers for RCC [3]. And Tao constructed a immune-related gene-based prognostic index, which can effectively predict the prognosis of patients with renal cancer and the associated immunoinfiltrating cells and provide a new method for predicting the prognosis and targeted therapy of renal cancer [7].

Based on the above researches and methods, the author analyzed the gene expression profile of ccRCC by using the GEO database, and then further analyzed the data to provide valuable hub genes for the following translational and clinical research.

## Materials and methods

### Access to public resources

Three expression profiling datasets (GSE14762 [8], GSE66270 [9] and GSE53757 [10]) were downloaded from the Gene Expression Omni - bus (GEO) database of the National Center for Biotechnology Information (NCBI). The GSE14762 dataset included 11 tumor tissue samples and matched normal tissue samples. The GSE66270 dataset included 14 normal tissue samples and 14 tumor tissue samples. And the GSE53757 dataset included 72 tumor tissue samples and adjacent tissue samples. Among of them, the microarray data of GSE14762 was running at the GPL4866 Plaforms, and the microarray data of GSE66270 and GSE53757 were analyzed at the GPL570 Plaforms. Platforms and series matrix files were downloaded as TXT files. Details for GEO ccRCC data were shown in Table 1.

**Table 1:** Details for GEO ccRCC data

Reference	GEO	Platform	Sample	
			normal	tumor
Furge K [8]^1^	GSE14762	GPL4866	11	11
Jung K [9]^2^	GSE66270	GPL570	14	14
Von Roemeling CA [10]^3^	GSE53757	GPL570	72	72

^1^. Renal Cell Carcinoma: Hypoxia and Endocytosis

^2^. Genome-wide analysis of gene expression patterns in human kidney cancer [patients without metastasis

^3^. Gene array analysis of clear cell renal cell carcinoma tissue versus matched normal kidney tissue

### Detection of DEGs

The R language software (version 3.5.0; https://www.r-project.org/) and annotation package were used to handle the downloaded data files. Probe name in the downloaded data files was changed into international standard name. The package in the Bioconductor (http://www.bioconductor.org/) was used for gene distinguish expression analysis. Robust Multi-array Average (RMA) algorithm was used for the gene expression profile data preprocessing. And quantile normalization was performed to normalize the above data. *P < 0.05* and *[log2 Fold Change] ≥ 2* were regarded as the DEGs screening threshold. The Robust Rank Aggreg (RRA) analysis (http://cran.r-project.org/) was used to list the up-regulated and down-regulated genes. DEGs of three datasets were represented by volcano map and hierarchical clustering heat map.

### Gene ontology (GO) and KEGG enrichment analyses

The biological processes (BP), molecular functions (MF) and cellular components (CC) of DEGs were explored by applying two online biological tools. The online website g:Profiler (https://biit.cs.ut.ee/gprofiler/gost) was used for Go analysis. And DAVID 6.8 (https://david.ncifcrf.gov/) was used for KEGG analysis. *P < 0.05* was considered as the significant threshold for GO and KEGG pathway analysis.

### PPI network construction

Online database STRING (http://string-db.org) and Cytoscape software (Version 3.6.1, http://www.cytoscape.org/) were applied to generate the PPI network of DEGs and identify the hub genes. Besides, the Molecular Complex Detection (MCODE) plug-in in Cytoscape software was used to analyze clustered sub-networks of highly intraconnected nodes from the above PPI network. The default parameters of MCODE plug-in were as follows: Degree cutoff ≥2, Node score cutoff ≥0.2, K-core ≥2, and Max depth = 100.

### Expression and survival analysis of hub genes

The meta-analysis function of oncomine database (https://www.oncomine.org/) was used to better validate the expression level of hub genes. Besides, online database GEPIA (http://gepia.cancer-pku.cn/detail.php) was an interactive web server, which can analyze the expression of tumor and normal genes. The purpose of this study was to analyze the relationship between the hub genes expression and the survival analysis of [overall survival (OS) and disease free survival (DFS)].

## Results

### Microarray data information

**Fig. 1:**
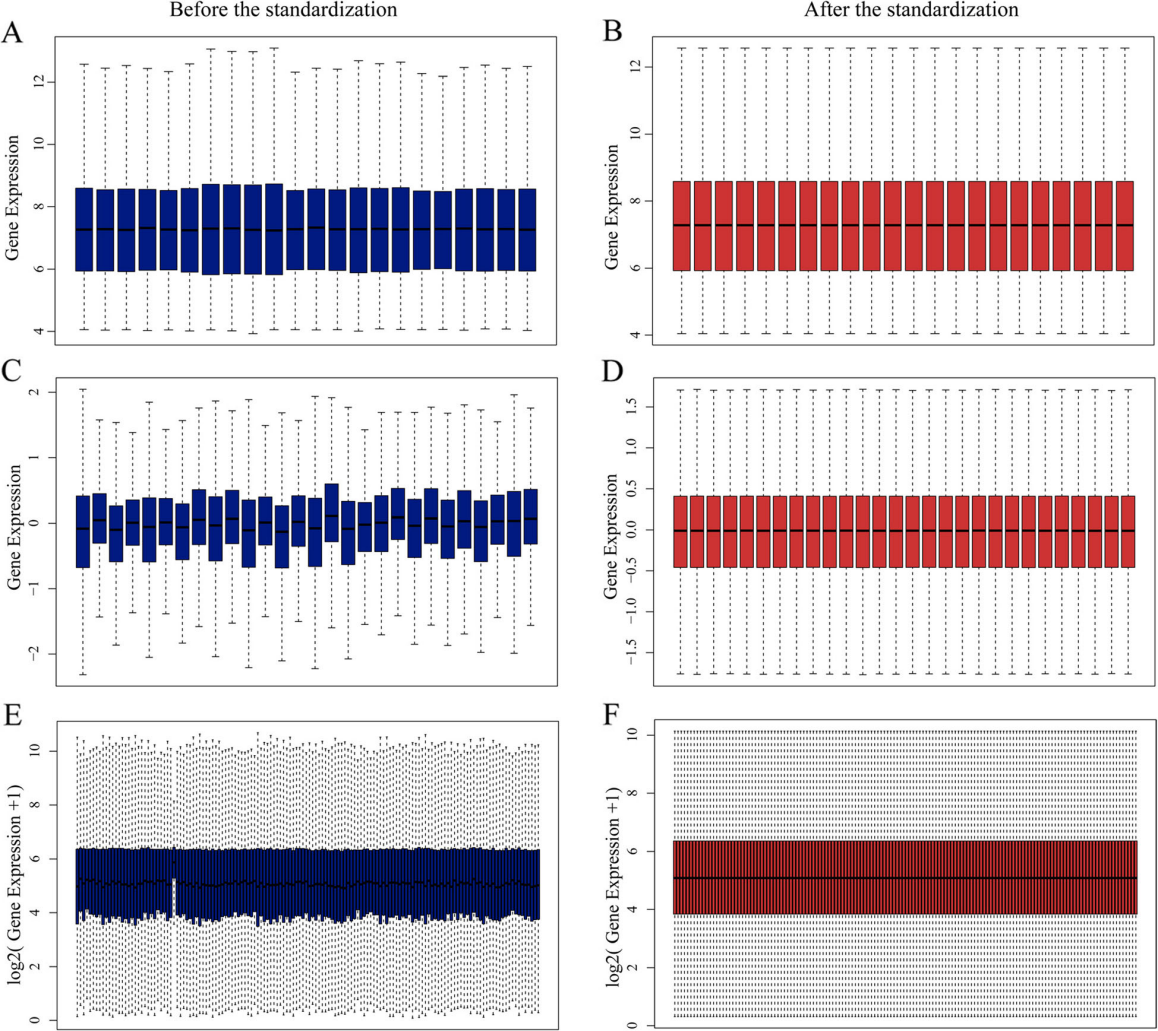
Standardization of gene expression by boxplot. The GSE14762 data (**A**), GSE66270 data (**B**) and GSE55757 data (**C**) was standardized

**Fig. 2:**
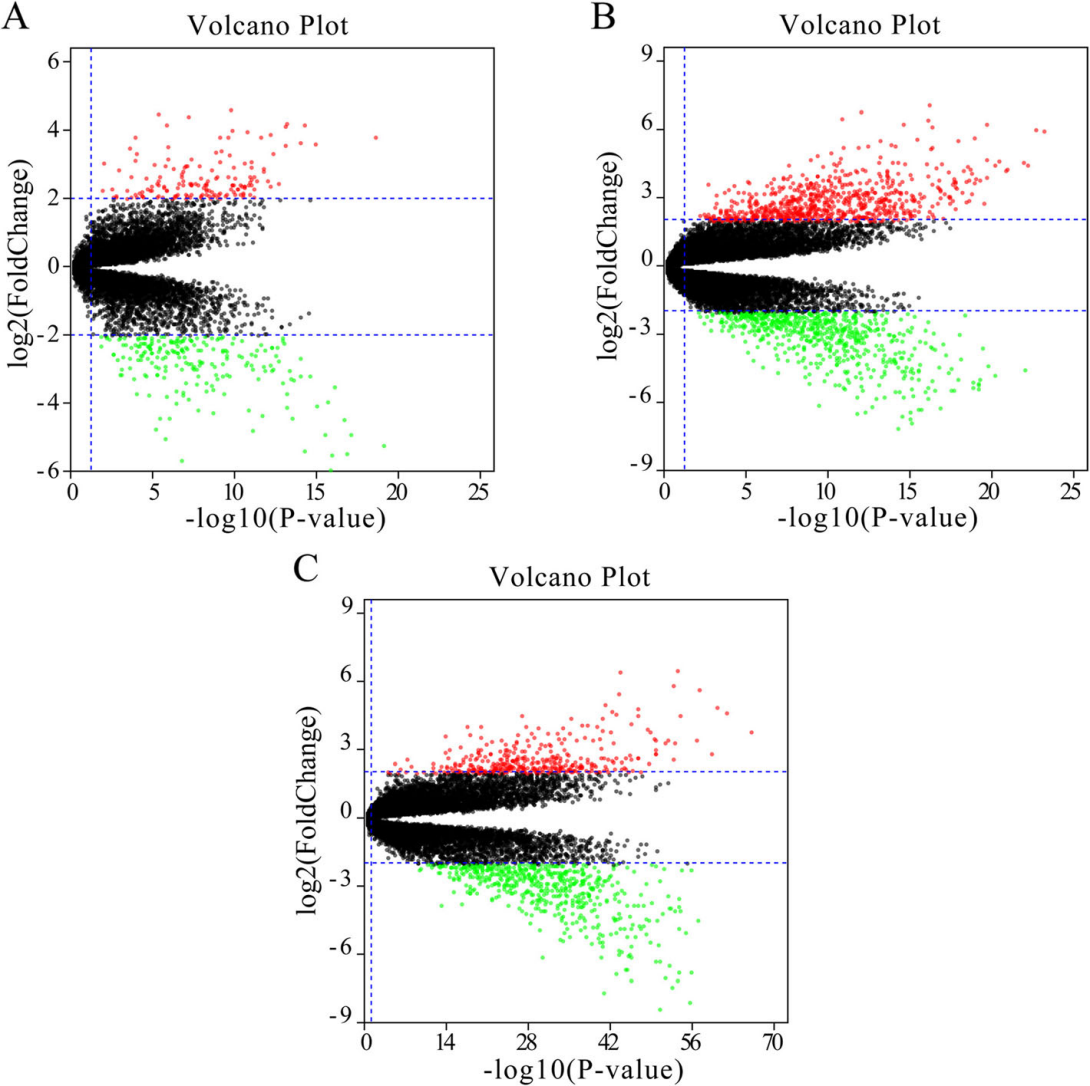
Volcano plot of differential data expressions between two sample sets. Three figures show the volcano plot of GSE14762 data (**A**), GSE66270 data (**B**) and GSE55757 data (**C**). The red oints represent overexpressed genes (threshold: *P < 0.05* and *|[log2 FC]| ≥ 2*). The green points represent under-expressed genes (threshold: *P < 0.05* and *|[log2 FC]| ≥ 2*). The black points represent undifferentiated genes

**Fig. 3:**
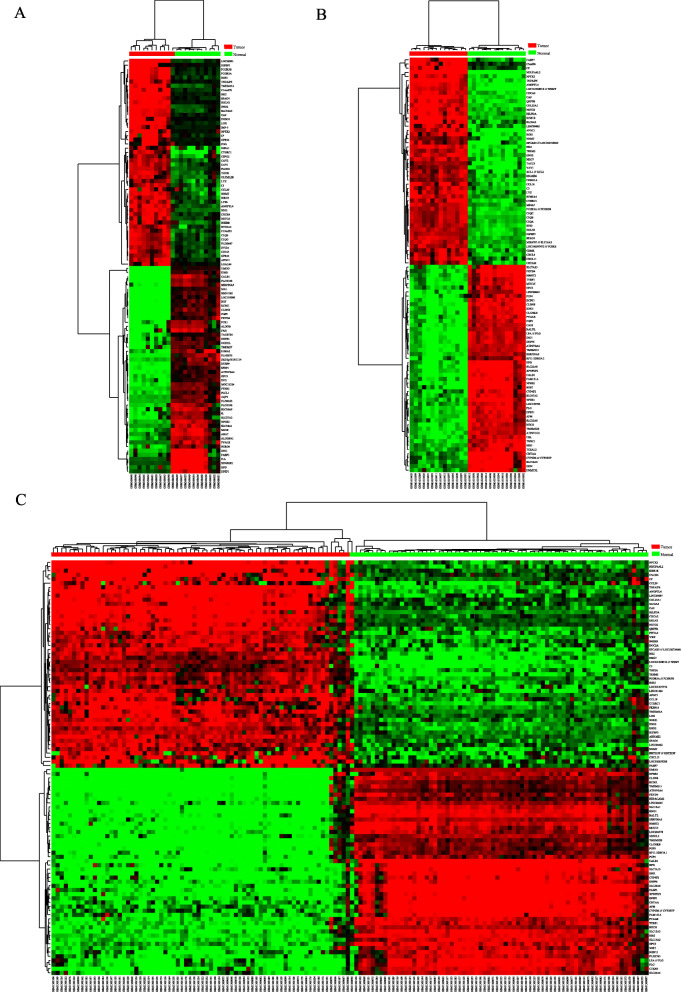
Clustering heatmap of DEGs. Three figures show the heatmap of GSE14762 data (**A**), GSE66270 data (**B**) and GSE55757 data (**C**). Red grid shows that the genes expression is uoverexpressed, green grid shows that the genes expression is under-expressed, black grid shows that there are no significant difference and gray grid shows that genes are too weak to be detected

The RCC expression microarray datasets (GSE14762, GSE66270 and GSE53757) were standardized by RMA algorithm, and the results were shown in Fig. 1. The author obtained 381 DREs from GSE14762 in accordance with the screening criteria (*P* < 0.05 and [log2 FC] ≥ 2). Moreover, the author obtained 870 DEGs and 1324 DEGs from GSE66270 and GSE53757. The DEGs from the two groups of sample data included in each of the three databases were shown by volcano plot (Fig. 2). The cluster heatmaps of the top 100 DEGs from the three microarrays were shown in Fig. 3.

### DEGs identification in ccRCC

**Fig. 4:**
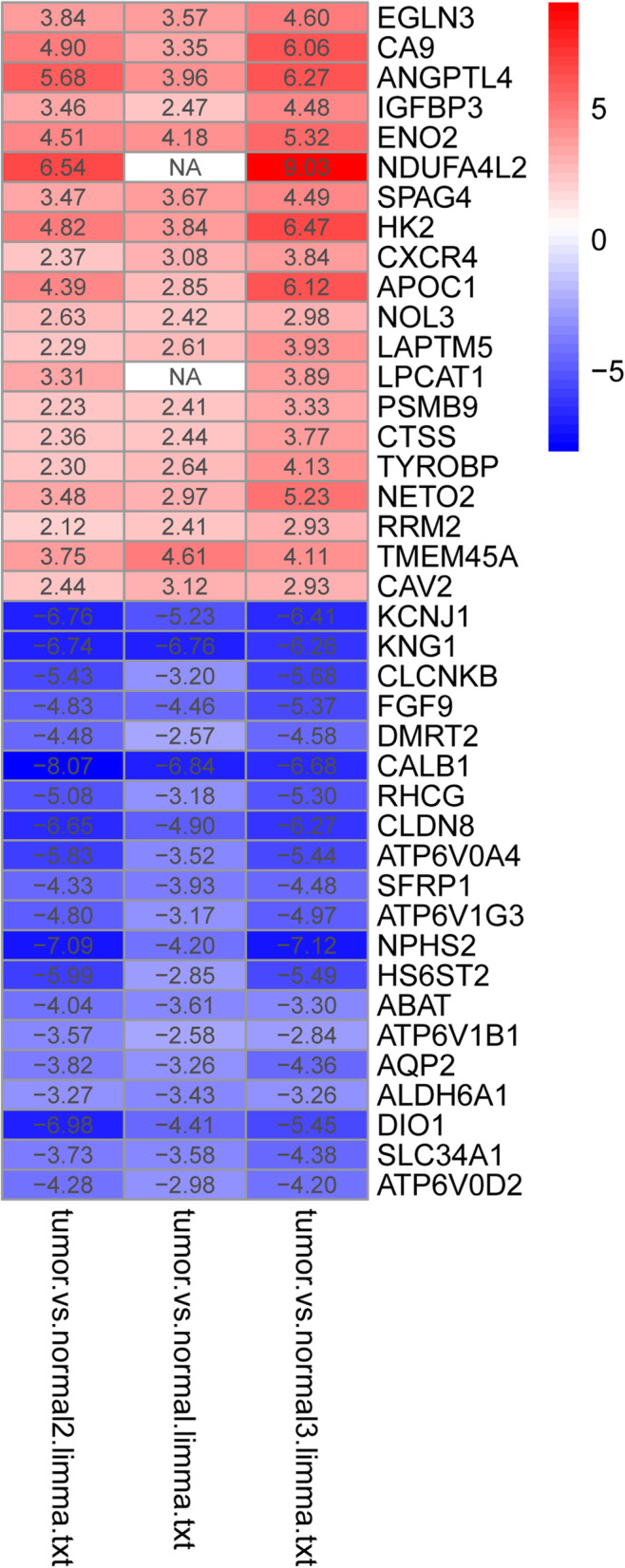
RRA analyses. This figure shows the top 20 overexpressed and under-expressed genes obtained by RRA analyses. Red grid indicates that the genes expression is upregulated, blue grid indicates that the genes expression is downregulated, and white grid indicates that there is no detected gene expression

The three microarray databases of RCC were analyzed and sorted by the limma package (threshold: *P < 0.05* and *[log2 Fold Change] ≥ 2*), and then further analyzed by the RRA method. As a result, 137 DEGs were identified, including 63 overexpressed genes and 74 under-expressed genes (Table 2). The heatmap of the top 20 overexpressed and under-expressed genes was revealed by R-heatmap software in Fig. 4.

**Table 2:** The genes differentially expressed both in GEO database were identified in ccRCC samples

	Gene names
Upregulated DEGs	EGLN3, CA9, ANGPTL4, IGFBP3, ENO2, NDUFA4L2, SPAG4, HK2, CXCR4, APOC1, NOL3, LAPTM5, LPCAT1, PSMB9, CTSS, TYROBP, NETO2, RRM2, TMEM45A, CAV2, LOC101928916 /// NNMT, TNFAIP6, PFKP, TLR3, LGALS1, MIR6787 /// SLC16A3, C3, COL23A1, C1QA, CSTA, CAV1, ITGB2, SEMA5B, PLOD2, C1QB, TRIB3, MS4A6A, PDK1, BIRC3, DDB2, ENTPD1, TREM2, EVI2A, P2RX7, HILPDA, LOC56901, FBXO16 /// ZNF395, ST8SIA4, CTHRC1, PRKCDBP, ENPP3, ISG20, MNDA, SLC16A3, ZNF395, FCER1G, PLK2, TNFSF13B, FCGR3A /// FCGR3B, RGS1, TLR2, TGFBI, CASP1
Downregulated DEGs	KCNJ1, KNG1, CLCNKB, FGF9, DMRT2, CALB1, RHCG, CLDN8, ATP6V0A4, SFRP1, ATP6V1G3, NPHS2, HS6ST2, ABAT, ATP6V1B1, AQP2, ALDH6A1, DIO1, SLC34A1, ATP6V0D2, RHBG, MAN1C1, FGF1, PVALB, UMOD, GPC3, DPEP1, SERPINA5, XPNPEP2, DCXR, TMEM52B, ACOX2, TMEM213, LPPR1, HEPACAM2, GPR110, TFCP2L1, FXYD4, HRG, GGT6, ERP27, SLC12A3, TYRP1, DUSP9, SH3GL2, SMIM5, SUCLG1, UPP2, SLC4A1, SLC22A8, SLC7A8, HSD11B2, ACAA1, SOST, ENPP6, RP11-999E24.3, ALDH4A1 TCF21, EFHD1, FBP1, HPD, TMEM30B, SLC13A3, SLC22A7, AFM, ACSF2, PCK2, PLG, FABP1, LOC155006, SUCNR1, LINC01187, CRYAA, CHL1

### GO and KEGG analysis of DEG

**Fig. 5:**
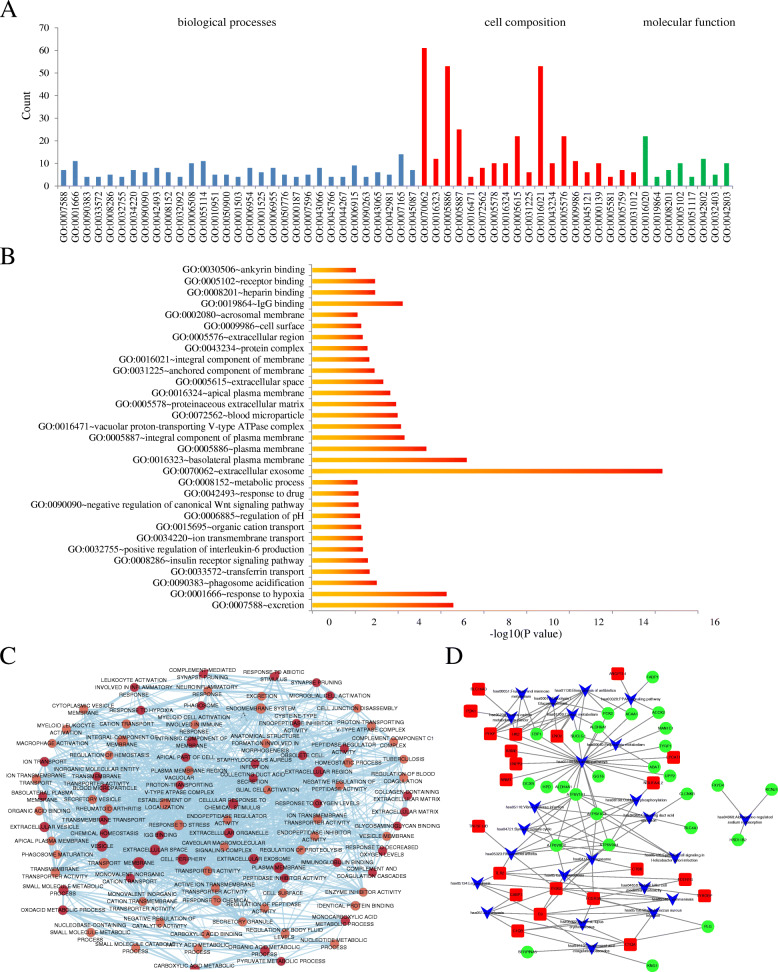
Go and KEGG analysis of DREs in ccRCC. **A** DEGs were divided into three functional groups by GO analysis, including molecular function, biological processes and cell composition. **B** GO enrichment significance items of DEGs in three functional groups. **C** The relationship between the different functions. **D** Significant pathway enrichment of DEGs. Purple represents the signaling pathway, red represents the overexpressed genes and green represents the under-expressed genes

The author futher understood the function of hub genes include BP, CC and MF by using DAVID database. Significant results of the GO enrichment analysis of DEGs in ccRCC are shown in Table 3. As shown in Fig. 5a and b, GO analysis (threshold: *P* < 0.05 and count≥2) demonstrated that ccRCC hub genes were mainly enriched in 50 terms of BP group, such as response to hypoxia, oxidation-reduction process and proteolysis. In CC group, DEGs were enriched in 21 terms, such as extracellular exosome, plasma membrane and membrane integral component. Similarly in MF group, DEGs were enriched in 11 terms, such as identical protein binding, receptor binding and heparin binding. As shown in Fig. 5c, the result illustrated the relationship between the different functions of cytoscape software.

**Table 3:** Significant results of the GO enrichment analysis of DEGs

Function	Term	Count	PValue	Genes
biological processes	GO:0007588 ~ excretion	7	2.64E-07	NPHS2, CLCNKB, UMOD, ATP6V1B1, ATP6V0A4, KCNJ1, AQP2
	GO:0001666 ~ response to hypoxia	11	5.42E-07	CAV1, NOL3, CA9, PLOD2, CXCR4, EGLN3, TLR2, HSD11B2, ABAT, CASP1, ANGPTL4
	GO:0090383 ~ phagosome acidification	4	9.87E-04	ATP6V1G3, ATP6V1B1, ATP6V0A4, ATP6V0D2
	GO:0033572 ~ transferrin transport	4	0.002115	ATP6V1G3, ATP6V1B1, ATP6V0A4, ATP6V0D2
	GO:0008286 ~ insulin receptor signaling pathway	5	0.002575	CAV2, ATP6V1G3, ATP6V1B1, ATP6V0A4, ATP6V0D2
	GO:0032755 ~ positive regulation of interleukin-6 production	4	0.004349	P2RX7, TLR2, FCER1G, TLR3
	GO:0034220 ~ ion transmembrane transport	7	0.004506	FXYD4, CLCNKB, ATP6V1G3, ATP6V1B1, ATP6V0A4, ATP6V0D2, AQP2
	GO:0015695 ~ organic cation transport	3	0.00525	RHCG, SLC7A8, RHBG
	GO:0006885 ~ regulation of pH	3	0.005972	RHCG, ATP6V1B1, ATP6V0A4
	GO:0090090 ~ negative regulation of canonical Wnt signaling pathway	6	0.006989	CTHRC1, CAV1, SOST, GPC3, SFRP1, PSMB9
	GO:0042493 ~ response to drug	8	0.007086	P2RX7, CA9, SFRP1, LGALS1, SLC34A1, HSD11B2, ABAT, NNMT
	GO:0008152 ~ metabolic process	6	0.007913	ENPP6, LPCAT1, SUCLG1, MAN1C1, ACSF2, ACAA1
	GO:0032092 ~ positive regulation of protein binding	4	0.010136	CTHRC1, CAV1, PLK2, TRIB3
	GO:0055074 ~ calcium ion homeostasis	3	0.011171	CAV1, ATP6V1B1, CALB1
	GO:0006508 ~ proteolysis	10	0.011293	C1QA, C1QB, GGT6, SFRP1, C3, CTSS, CASP1, PLG, DPEP1, XPNPEP2
	GO:0055114 ~ oxidation-reduction process	11	0.011711	ALDH6A1, TYRP1, PLOD2, NDUFA4L2, RRM2, EGLN3, HSD11B2, ALDH4A1, DIO1, DCXR, HPD
	GO:0010951 ~ negative regulation of endopeptidase activity	5	0.012067	KNG1, C3, SERPINA5, HRG, CSTA
	GO:0050900 ~ leukocyte migration	5	0.012407	SLC16A3, CAV1, SLC7A8, FCER1G, ITGB2
	GO:0045880 ~ positive regulation of smoothened signaling pathway	3	0.013221	GPC3, SFRP1, FGF9
	GO:0001798 ~ positive regulation of type IIa hypersensitivity	2	0.014597	C3, FCER1G
	GO:2000054 ~ negative regulation of Wnt signaling pathway involved in dorsal/ventral axis specification	2	0.014597	SOST, SFRP1
	GO:0061621 ~ canonical glycolysis	3	0.01542	ENO2, PFKP, HK2
	GO:0001503 ~ ossification	4	0.020939	SOST, SLC34A1, ATP6V1B1, ATP6V0A4
	GO:0006954 ~ inflammatory response	8	0.021562	KNG1, TNFAIP6, P2RX7, CXCR4, C3, TLR2, TLR3, ITGB2
	GO:0050717 ~ positive regulation of interleukin-1 alpha secretion	2	0.021815	P2RX7, CASP1
	GO:2000116 ~ regulation of cysteine-type endopeptidase activity	2	0.021815	BIRC3, PSMB9
	GO:0070634 ~ transepithelial ammonium transport	2	0.021815	RHCG, RHBG
	GO:0019065 ~ receptor-mediated endocytosis of virus by host cell	2	0.021815	CAV2, CAV1
	GO:0015991 ~ ATP hydrolysis coupled proton transport	3	0.022874	ATP6V1B1, ATP6V0A4, ATP6V0D2
	GO:0051480 ~ regulation of cytosolic calcium ion concentration	3	0.022874	CAV1, PVALB, CALB1
	GO:0001525 ~ angiogenesis	6	0.024169	CAV1, FGF9, TGFBI, HRG, FGF1, ANGPTL4
	GO:0002931 ~ response to ischemia	3	0.024234	CAV1, NOL3, HK2
	GO:0072221 ~ metanephric distal convoluted tubule development	2	0.028982	UMOD, CALB1
	GO:0002283 ~ neutrophil activation involved in immune response	2	0.028982	FCER1G, TYROBP
	GO:0007162 ~ negative regulation of cell adhesion	3	0.029996	KNG1, TGFBI, HRG
	GO:0006955 ~ immune response	8	0.03533	RGS1, TNFSF13B, C3, ENPP3, TLR2, CTSS, FCGR3A, FCGR3B
	GO:0070836 ~ caveola assembly	2	0.036096	CAV2, CAV1
	GO:0015696 ~ ammonium transport	2	0.036096	RHCG, RHBG
	GO:0006873 ~ cellular ion homeostasis	2	0.036096	RHCG, SLC4A1
	GO:0019740 ~ nitrogen utilization	2	0.036096	RHCG, RHBG
	GO:0010543 ~ regulation of platelet activation	2	0.036096	FCER1G, HRG
	GO:0051005 ~ negative regulation of lipoprotein lipase activity	2	0.036096	APOC1, ANGPTL4
	GO:0034123 ~ positive regulation of toll-like receptor signaling pathway	2	0.036096	TLR2, TLR3
	GO:0031623 ~ receptor internalization	3	0.039536	CAV1, FCER1G, ITGB2
	GO:0006094 ~ gluconeogenesis	3	0.041225	ENO2, FBP1, PCK2
	GO:0050776 ~ regulation of immune response	5	0.041952	C3, ITGB2, FCGR3A, TREM2, TYROBP
	GO:0030514 ~ negative regulation of BMP signaling pathway	3	0.04294	CAV1, SOST, SFRP1
	GO:0000187 ~ activation of MAPK activity	4	0.043944	P2RX7, CXCR4, FGF1, DUSP9
	GO:0007596 ~ blood coagulation	5	0.046407	P2RX7, SERPINA5, FCER1G, ENTPD1, PLG
	GO:0032760 ~ positive regulation of tumor necrosis factor production	3	0.046448	TLR2, FCER1G, TLR3
cell composition	GO:0070062 ~ extracellular exosome	61	4.70E-17	FGF9, SLC7A8, CALB1, AQP2, EFHD1, GPC3, PVALB, CXCR4, PLOD2, SERPINA5, TMEM52B, TGFBI, SLC4A1, FCGR3A, ATP6V0D2, FCGR3B, DPEP1, HPD, KNG1, ALDH6A1, CRYAA, SUCLG1, SLC22A8, PFKP, FBP1, C1QA, C1QB, RHCG, MNDA, ABAT, CSTA, CHL1, SH3GL2, ENPP6, C3, ENPP3, APOC1, ITGB2, UMOD, ATP6V1B1, ENO2, HS6ST2, HRG, SUCNR1, ENTPD1, SLC12A3, LGALS1, PCK2, MAN1C1, PLG, PSMB9, XPNPEP2, AFM, GGT6, SFRP1, NPHS2, SLC13A3, FABP1, ATP6V0A4, IGFBP3, DCXR
	GO:0016323 ~ basolateral plasma membrane	12	6.23E-08	CLDN8, CAV1, RHCG, CA9, SLC22A7, SLC22A8, SLC7A8, RHBG, UMOD, SLC4A1, ATP6V1B1, AQP2
	GO:0005886 ~ plasma membrane	53	4.79E-06	CLDN8, TLR2, SLC7A8, AQP2, GPC3, CXCR4, TGFBI, SLC4A1, FCGR3A, FCGR3B, DPEP1, KNG1, COL23A1, SLC22A7, SUCLG1, SLC22A8, SLC34A1, TNFSF13B, RHCG, CA9, ATP6V1G3, TREM2, SH3GL2, CHL1, ENPP6, CAV2, CAV1, FXYD4, C3, RHBG, TRIB3, CLCNKB, ITGB2, KCNJ1, ENO2, TMEM30B, FCER1G, HRG, SUCNR1, ENTPD1, TYROBP, SLC12A3, PLG, XPNPEP2, SLC16A3, P2RX7, RGS1, SFRP1, NPHS2, SLC13A3, DIO1, ATP6V0A4, DCXR
	GO:0005887 ~ integral component of plasma membrane	25	5.00E-05	CAV2, CAV1, FXYD4, SLC12A3, SLC22A7, ENPP3, SLC22A8, TLR2, RHBG, SLC34A1, SLC7A8, TLR3, CLCNKB, AQP2, SLC16A3, P2RX7, LAPTM5, GPC3, RHCG, NPHS2, FCER1G, SLC13A3, SLC4A1, ENTPD1, TYROBP
	GO:0016471 ~ vacuolar proton-transporting V-type ATPase complex	4	7.28E-05	ATP6V1G3, ATP6V1B1, ATP6V0A4, ATP6V0D2
	GO:0072562 ~ blood microparticle	8	1.04E-04	KNG1, C1QB, AFM, C3, HRG, SLC4A1, PLG, ANGPTL4
	GO:0005578 ~ proteinaceous extracellular matrix	10	1.24E-04	CTHRC1, SOST, GPC3, SFRP1, LGALS1, TGFBI, UMOD, FGF1, CHL1, ANGPTL4
	GO:0016324 ~ apical plasma membrane	10	2.30E-04	CAV1, RHCG, SLC12A3, SLC34A1, UMOD, ATP6V1B1, ATP6V0A4, ATP6V0D2, DPEP1, AQP2
	GO:0005615 ~ extracellular space	22	4.94E-04	KNG1, CTHRC1, C3, FGF9, LGALS1, HILPDA, CTSS, PLG, TNFAIP6, AFM, GPC3, SOST, TNFSF13B, SFRP1, SERPINA5, TGFBI, ENO2, CSTA, FGF1, IGFBP3, DPEP1, ANGPTL4
	GO:0031225 ~ anchored component of membrane	6	0.001254	ENPP6, GPC3, UMOD, FCGR3B, DPEP1, XPNPEP2
	GO:0016021 ~ integral component of membrane	53	0.002201	CLDN8, TLR2, SLC7A8, TLR3, AQP2, CXCR4, EVI2A, TMEM52B, SMIM5, SLC4A1, FCGR3A, FCGR3B, COL23A1, SLC22A7, SLC22A8, SLC34A1, DMRT2, HEPACAM2, TNFSF13B, CA9, RHCG, SPAG4, HSD11B2, TREM2, CHL1, NETO2, CAV2, TYRP1, CAV1, TMEM213, ENPP3, RHBG, CLCNKB, UMOD, KCNJ1, SEMA5B, LPCAT1, TMEM30B, FCER1G, HS6ST2, SUCNR1, ENTPD1, MS4A6A, TYROBP, TMEM45A, SLC12A3, NDUFA4L2, HILPDA, GGT6, SFRP1, SLC13A3, DIO1, ATP6V0A4
	GO:0043234 ~ protein complex	10	0.002667	CAV2, CAV1, SOST, PVALB, SERPINA5, NPHS2, DDB2, FABP1, PRKCDBP, BIRC3
	GO:0005576 ~ extracellular region	22	0.004463	KNG1, ENPP6, FGF9, C3, APOC1, UMOD, CTSS, PLG, C1QA, C1QB, AFM, SOST, TNFSF13B, SFRP1, SERPINA5, TGFBI, HRG, FGF1, TREM2, CASP1, IGFBP3, ANGPTL4
	GO:0009986 ~ cell surface	11	0.005205	SFRP1, CXCR4, LGALS1, TLR2, SLC34A1, FCER1G, TLR3, HILPDA, ITGB2, PLG, TYROBP
	GO:0002080 ~ acrosomal membrane	3	0.007858	CAV2, CAV1, SERPINA5
	GO:0005602 ~ complement component C1 complex	2	0.014107	C1QA, C1QB
	GO:0045121 ~ membrane raft	6	0.015662	CAV2, CAV1, NPHS2, TLR2, SLC34A1, BIRC3
	GO:0000139 ~ Golgi membrane	10	0.024687	CAV2, CAV1, LPCAT1, ST8SIA4, TLR3, HS6ST2, HEPACAM2, MAN1C1, SH3GL2, HPD
	GO:0005581 ~ collagen trimer	4	0.027574	C1QA, CTHRC1, C1QB, COL23A1
	GO:0005759 ~ mitochondrial matrix	7	0.028942	PDK1, ALDH6A1, SUCLG1, ALDH4A1, ABAT, PCK2, ACSF2
	GO:0005782 ~ peroxisomal matrix	3	0.042028	ACOX2, FABP1, ACAA1
molecular function	GO:0019864 ~ IgG binding	4	6.11E-05	FCER1G, UMOD, FCGR3A, FCGR3B
	GO:0008201 ~ heparin binding	7	0.001175	KNG1, SOST, SFRP1, FGF9, SERPINA5, HRG, FGF1
	GO:0005102 ~ receptor binding	10	0.001189	KNG1, ACOX2, P2RX7, CAV1, TNFSF13B, C3, HRG, HILPDA, PLG, TYROBP
	GO:0030506 ~ ankyrin binding	3	0.009327	RHCG, RHBG, SLC4A1
	GO:0001530 ~ lipopolysaccharide binding	3	0.011232	P2RX7, TLR2, TREM2
	GO:0043027 ~ cysteine-type endopeptidase inhibitor activity involved in apoptotic process	3	0.012243	NOL3, BIRC3, DPEP1
	GO:0015301 ~ anion:anion antiporter activity	3	0.012243	SLC22A7, SLC22A8, SLC4A1
	GO:0051117 ~ ATPase binding	4	0.017177	CAV1, FXYD4, ATP6V1G3, ATP6V0A4
	GO:0042802 ~ identical protein binding	12	0.022021	CLDN8, CAV1, NOL3, SFRP1, CRYAA, FBP1, ALDH4A1, TLR3, SH3GL2, DCXR, TYROBP, ANGPTL4
	GO:0015078 ~ hydrogen ion transmembrane transporter activity	3	0.022995	ATP6V1B1, ATP6V0A4, ATP6V0D2
	GO:0004869 ~ cysteine-type endopeptidase inhibitor activity	3	0.025762	KNG1, HRG, CSTA

The significantly enriched pathways were submitted to KEGG analysis to further analyze the above DEGs. As shown in Table 4 and Fig. 5d, the significant pathway enrichment of DEGs was indicated by KEGG analysis. These DEGs were enriched in 24 pathways, which mainly related to metabolic pathways, phagosome and other pathways.

**Table 4:** KEGG pathway analysis of DEGs associated with ccRCC

Term	Count	*P* Value	Genes
hsa04966:Collecting duct acid secretion	6	1.77E-05	CLCNKB, SLC4A1, ATP6V1G3, ATP6V1B1, ATP6V0A4, ATP6V0D2
hsa05150:Staphylococcus aureus infection	7	5.12E-05	C1QA, C1QB, C3, ITGB2, FCGR3A, FCGR3B, PLG
hsa04145:Phagosome	10	9.64E-05	C3, TLR2, ITGB2, CTSS, ATP6V1G3, FCGR3A, ATP6V1B1, ATP6V0A4, FCGR3B, ATP6V0D2
hsa05323:Rheumatoid arthritis	7	7.65E-04	TNFSF13B, TLR2, ITGB2, ATP6V1G3, ATP6V1B1, ATP6V0A4, ATP6V0D2
hsa05152:Tuberculosis	9	0.001573	C3, TLR2, FCER1G, ITGB2, CTSS, FCGR3A, ATP6V0A4, FCGR3B, ATP6V0D2
hsa04610:Complement and coagulation cascades	6	0.001629	KNG1, C1QA, C1QB, C3, SERPINA5, PLG
hsa01100:Metabolic pathways	26	0.006292	ACOX2, TYRP1, ENPP3, HK2, UPP2, ATP6V1B1, LPCAT1, ENO2, ALDH4A1, ATP6V0D2, HPD, ALDH6A1, NDUFA4L2, SUCLG1, FBP1, PFKP, PCK2, MAN1C1, GGT6, RRM2, ABAT, ATP6V1G3, ATP6V0A4, DCXR, ACAA1, NNMT
hsa03320:PPAR signaling pathway	5	0.009598	ACOX2, FABP1, PCK2, ACAA1, ANGPTL4
hsa00010:Glycolysis / Gluconeogenesis	5	0.009598	ENO2, FBP1, PFKP, HK2, PCK2
hsa05140:Leishmaniasis	5	0.011719	C3, TLR2, ITGB2, FCGR3A, FCGR3B
hsa01200:Carbon metabolism	6	0.013278	ALDH6A1, SUCLG1, ENO2, FBP1, PFKP, HK2
hsa05133:Pertussis	5	0.01412	C1QA, C1QB, C3, ITGB2, CASP1
hsa05110:Vibrio cholerae infection	4	0.026859	ATP6V1G3, ATP6V1B1, ATP6V0A4, ATP6V0D2
hsa05134:Legionellosis	4	0.029616	C3, TLR2, ITGB2, CASP1
hsa04721:Synaptic vesicle cycle	4	0.043794	ATP6V1G3, ATP6V1B1, ATP6V0A4, ATP6V0D2
hsa05230:Central carbon metabolism in cancer	4	0.045546	SLC16A3, PDK1, PFKP, HK2
hsa00640:Propanoate metabolism	3	0.047335	ALDH6A1, SUCLG1, ABAT
hsa01130:Biosynthesis of antibiotics	7	0.04889	SUCLG1, ENO2, FBP1, PFKP, HK2, PCK2, ACAA1
hsa05120:Epithelial cell signaling in Helicobacter pylori infection	4	0.051005	ATP6V1G3, ATP6V1B1, ATP6V0A4, ATP6V0D2
hsa00051:Fructose and mannose metabolism	3	0.060164	FBP1, PFKP, HK2
hsa04650:Natural killer cell mediated cytotoxicity	5	0.065949	FCER1G, ITGB2, FCGR3A, FCGR3B, TYROBP
hsa00190:Oxidative phosphorylation	5	0.084557	NDUFA4L2, ATP6V1G3, ATP6V1B1, ATP6V0A4, ATP6V0D2
hsa04960:Aldosterone-regulated sodium reabsorption	3	0.085035	FXYD4, HSD11B2, KCNJ1
hsa05322:Systemic lupus erythematosus	5	0.086365	C1QA, C1QB, C3, FCGR3A, FCGR3B

### PPI network and module analysis

**Fig. 6:**
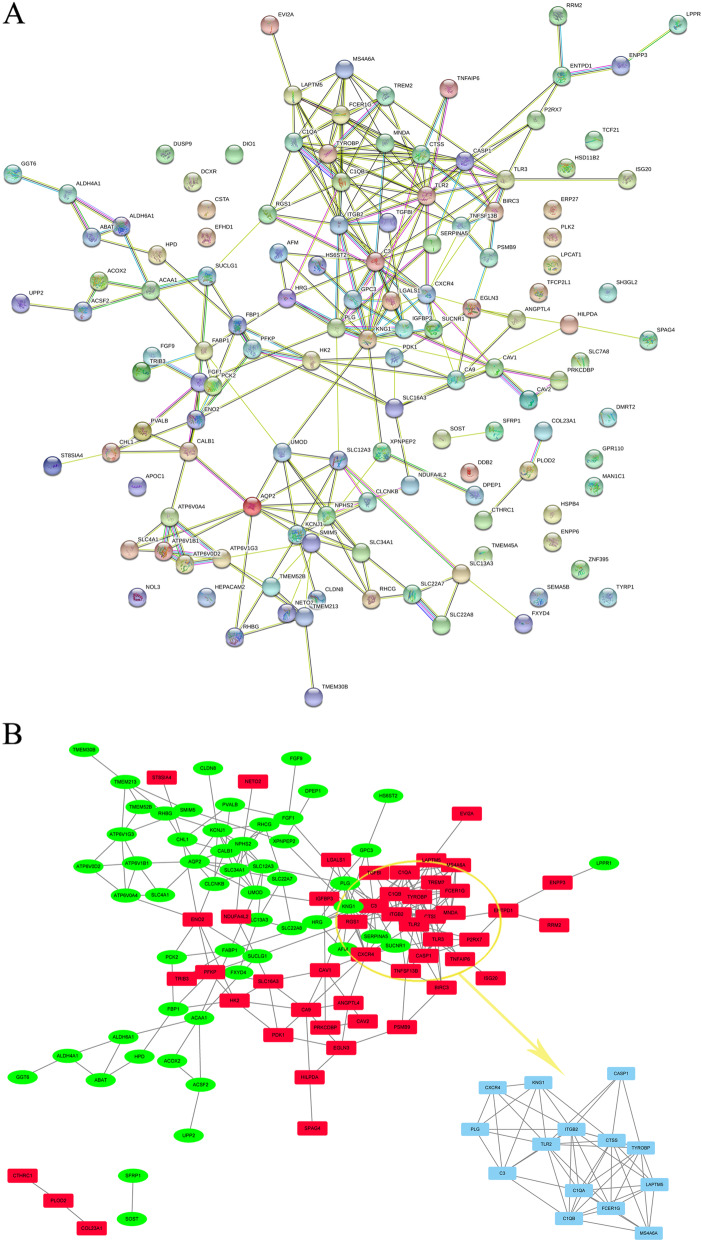
Protein-protein interaction network and MCODE application. **A** PPI network. **B** Top 13 degree genes by MCODE application

String database was used to generate PPI networks of DEGs in RCC. Figure 6a showed the relationship between the 137 candidate hub genes. Besides, MCODE application was applied to screen out the highest-scoring nodes. And Fig. 6b displayed the module with the highest score (score = 10, node = 11, edges = 50). As a result, MCODE application selected 13 nodes with the highest score, including 10 upregulated candidate genes (C1QA, C1QB, C3, CTSS, CXCR4, FCER1G, ITGB2, TLR2, TLR3 and TYROBP) and 3 downregulated candidate genes (AQP2, KNG1, PLG).

### Expression and survival analysis of hub genes

**Fig. 7:**
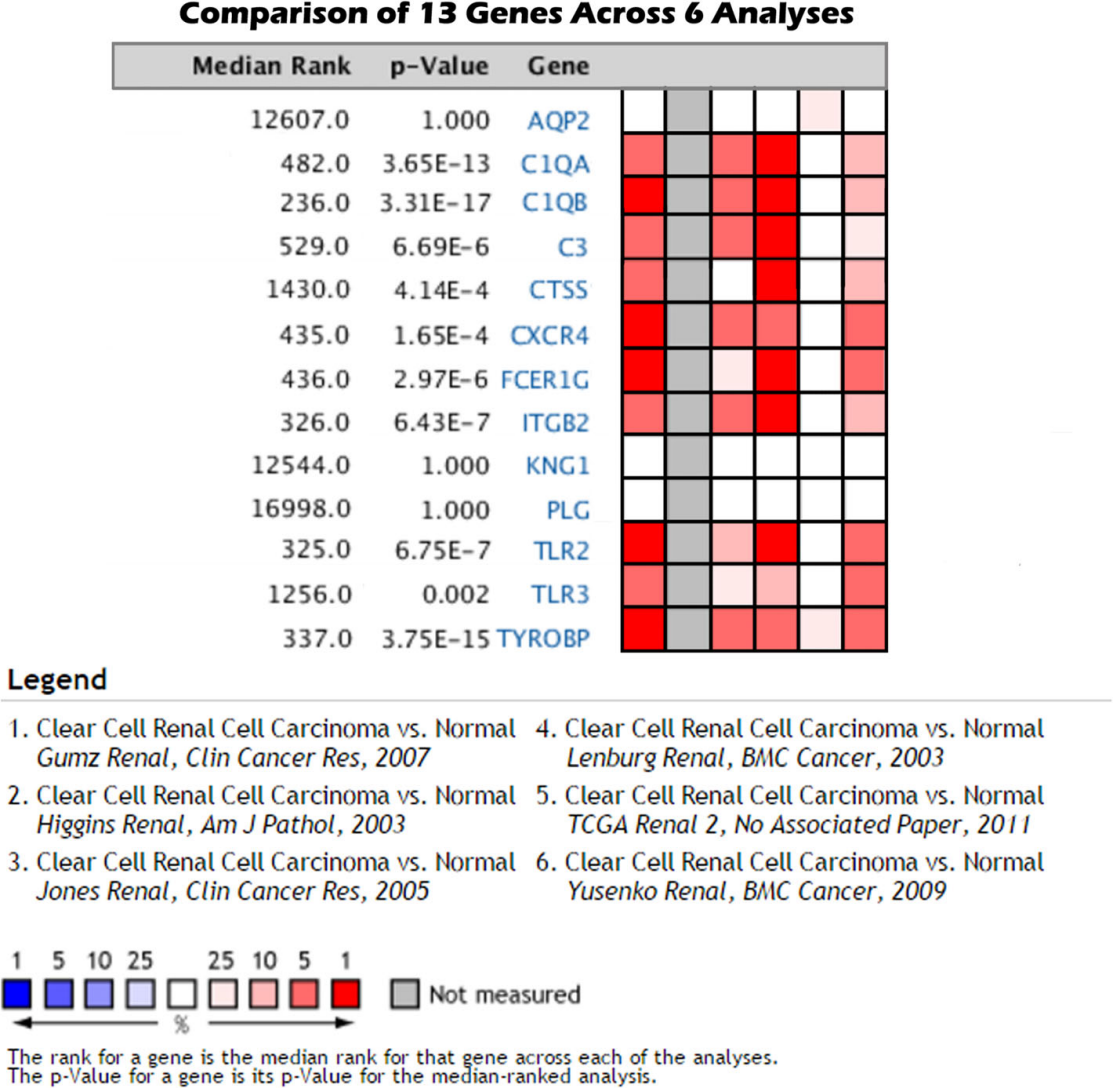
The expression level of 13 hub genes. Among 6 different analysis datasets by the ONCOMINE database

**Fig. 8:**
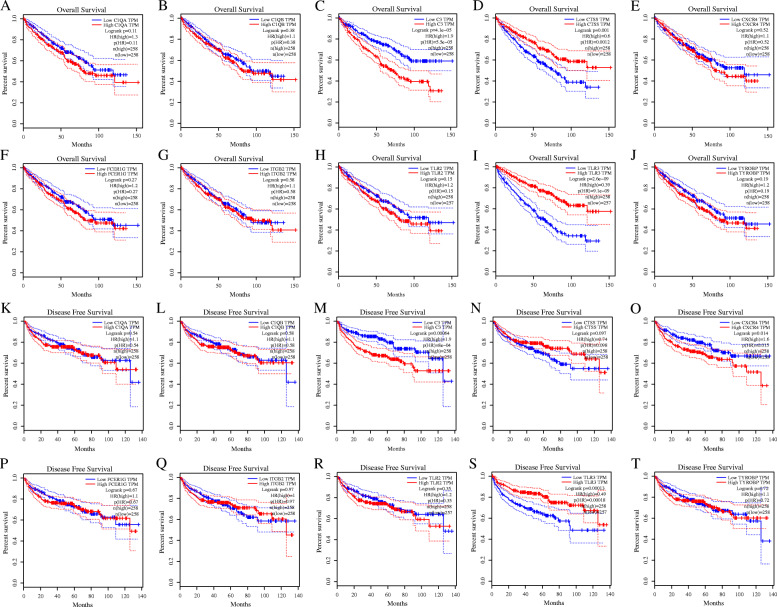
The OS and DFS of 10 candidate genes in ccRCC patients by GEPIA database. (OS: **A**-**J**, DFS: **K**-**T**)

The oncomine database and GEPIA database were applied to further explore the expression and prognosis of the above screened genes. Six analyses were obtained from the oncomine database (Fig. 7). The significant (*P < 0.05*) expression of 10 genes were suggested by the result of meta-analysis. Figure 8 indicated the OS and DFS of 10 genes. And the result demonstrated that ccRCC patients with high C3 expression had a poor OS, while ccRCC patients with high CTSS and TLR3 expressions had a good OS. Besides, in ccRCC patients, high C3 and CXCR4 expressions indicated a poor DFS, while high TLR3 expression indicated a good DFS. Finally, C3 and CXCR4 were selected to distinguish the prognosis of ccRCC patients.

## Discussion

Kidney cancer accounts for about 2 to 3% of adult malignant tumors, and 80 to 90% of adult renal malignancies. In 2012, about 338, 000 kidney cancer cases were newly discovered, accounting for 24% of all tumors; and there were 144,000 death cases, accounting for 17% of all tumors [11]. RCC was the most common kidney malignancies. The early symptoms of RCC were not obvious, and most patients are diagnosed with advanced stage or metastasis [12]. RCC was characteristic of easy recurrence and metastasis because of its complexity of the causes and pathogenesis. Moreover, it was insensitive to the traditional chemoradiotherapy. Under the influence of these reasons, RCC usually leaded to poor clinical outcomes. Hence, it could improve the diagnosis, treatment and prognosis of RCC via understanding more of the biological molecular mechanism.

The sequencing technology and bioinformatics are developing gradually, the collection and analysis of previous data will support to explor the pathogenesis of RCC and discover possible biomarkers for diagnosis andtreatment [13].

Bioinformatics method is a highly efficient research pathway, which could promote the development of related gene or group of disease by analyzing the biological data. At the present, Bioinformatics have been widely used at all areas, including medical research, the design of the discover disease-related genes, clinical diagnosis of disease, individualized treatment of diseases and new molecular targets for drug discovery [14].

One hundred thirty-seven DEGs were identified in this study, including 63 overexpressed genes and 74 under-expressed genes. It was found that these DEGs were mainly enriched in 82 terms and 24 pathways through GO and KEGG analysis. Thirteen highest-scoring genes were screened as hub gene through PPI network. Further verification based on the oncomine platform indicated that 10 hub genes (C1QA, C1QB, C3, CTSS, CXCR4, FCER1G, ITGB2, TLR2, TLR3 and TYROBP) had significantly highly expressed. Finally, through the GEPIA platform, the author found that ccRCC patients with high C3 expression had a poor OS, while ccRCC patients with high CTSS and TLR3 expressions had a better OS. Meanwhile, high C3 and CXCR4 expressions were associated with a poor DFS, while patients with high TLR3 expression had a good DFS.

As a protein coding gene, complement component 3 (C3) is involved in the occurrence and development of many diseases, including C3 deficiency, Autosomal Recessive and Hemolytic Uremic Syndrome, Atypical 5 [15]. And its related pathways are Immune response Lectin induced complement pathway and Signaling by GPCR. In previous reports, C3 was demonstrated as a potential prognostic marker for non-small cell lung cancer and may be a new immune marker to differentiate the prognosis of patients with non-small cell lung cancer [16, 17]. Besides, Yuan et al. demonstated that overexpressed C3 could activate the JAK2/STAT3 pathway, which affected the progression of gastric cancer [18]. In addition, it had been reported that tumor cell–derived C3 could regulated TAMs through C3a-C3aR-PI3Kγ pathway to suppress the antitumor immunity [19].

CTSS (Cathepsin S) is a protein coding gene. Previous articles in papillary thyroid carcinoma reported that CTSS was highly expressed and related to transformation. These results revealed that the highly expression of CTSS was associated with poor prognosis and lymph node metastasis [20]. Similarly, it had been reported that CTSS was over-expressed in triple-negative breast cancer, and the inhibition of CTSS could be conducted by inhibiting the growth and metastasis of triple-negative breast cancer [21]. Prof. Dheilly found that follicular lymphoma patients harbor a recurrent hotspot mutation targeting tyrosine 132 (Y132D) in cathepsin S (CTSS) that enhances protein activity. Futher study revealed that it could enhanced the anti-tumor immune responses in Non-Hodgkin Lymphoma by inhibiting CTSS [22]. In this study, the author analyzed the research data and found that CTSS was indeed highly expressed in RCC, but the high expression was associated with better prognosis. The prognosis of patients with high expression was even better, which is an opposite effect between expression and prognosis. The potential reasons for the inconsistent findings need further investigations.

As a member of the Toll-like receptor (TLR) family, previous studies had reported that TLR3 was abnormally expressed in a variety of tumors, including breast, ovarian and prostate tumors. But TLR3 was associated with the clinical outcomes of various cancers [23, 24]. Francesca revealed that TLR3 could induce apoptpsis in Non-Small-Cell Lung Cancer via boosting the innate immune response [25]. Besides, Fan’s result demonstated that TLR3 suppressed the proliferation by downregulating the EGFR/PI3K/AKT pathway in brest cancer [26]. Similarly, TLR3 was also downregulated in hepatocellular carcinoma. And deep reseach showed that overexpression of TLR3 was associated with longer survival [27]. In this study, TLR3 was highly expressed in RCC but it was related to the better prognosis result.

Chemokine receptor-4 (CXCR4) belongs to the super-family of the seven-transmembrane domain, heterotrimeric G-protein-coupled receptors and is associated with cell proliferation, migration, invasion and survival. In the previous reports, it had been demonstrated that CXCR4 was upregulated in sporadic Vestibular schwannomas (VS) as well as in neurofibromatosis type 2 (NF2) tumors [28–30]. Besides, SDF-1 (CXCL12)/CXCR4 signaling has been verified to play a vital role in oncobiology, especially in hypoxia adaptation, metastasis and migration [31]. What’s more, the CXCR4 antagonists (such as AMD3100, Mozobil®) were widely applied in hematopoietic stem cells, which could dramaticly increase the mobilization efficiency and yields of progenitor cells [32]. The results in this study showed that CXCR4 was over-expressed in RCC and associated with poor prognosis. However, the role of CXCR4 in RCC has been poorly studied. Therefore, the further exploration of the mechanism of CXCR4 in RCC will help people to find new therapeutic targets.

## Conclusion

In summary, the author identified two ccRCC-associated candidate genes (C3 and CXCR4) with potential prognostic value via bioinformatics analysis of three expression profile datasets from the GEO database. Additionally, in this study, it have been found that CTSS and TLR3 were abnormally expressed in ccRCC and associated with ccRCC prognosis. However, their expression level is contrary to the prognosis. These novel biomarkers may have important clinical significance for the diagnosis and prognosis of RCC, but their detailed action mechanism in the development of renal carcinoma needs to be further explored. In the following studies, the author will further verify the expression of the above genes in renal cancer through RT-QPCT. In addition, its downstream target genes and signaling pathways need to be explored and verified by cell experiments in vitro and animal experiments in vivo, which will help the author to better understand its developmental mechanism in renal cancer.

## Supplementary Information

**Additional file 1.**

**Additional file 2.**

## Data Availability

The data used to support the findings of this study are all included within the thesis. The gene expression data can be accessed on Gene Expression Omnibus (GEO). Oncomine database was used to get the expression profile of hub genes. GEPIA database was used to obtain the overall survival and disease free survival analysis of genes. The datasets analysed during this study are available in the: 1. GSE14762_RAW: https://www.ncbi.nlm.nih.gov/geo/download/?acc=GSE14762&format=file 2. GSE15641_RAW: https://www.ncbi.nlm.nih.gov/geo/download/?acc=GSE15641&format=file 3. GSE53757_RAW: https://www.ncbi.nlm.nih.gov/geo/download/?acc=GSE53757&format=file 4. GSE53757_series_matrix.txt: https://ftp.ncbi.nlm.nih.gov/geo/series/GSE53nnn/GSE53757/matrix/GSE53757_series_matrix.txt.gz
